# Shifts of Hydrogen Metabolism From Methanogenesis to Propionate Production in Response to Replacement of Forage Fiber With Non-forage Fiber Sources in Diets *in vitro*

**DOI:** 10.3389/fmicb.2018.02764

**Published:** 2018-11-15

**Authors:** Kun Wang, Xuemei Nan, Kangkang Chu, Jinjin Tong, Liang Yang, Shanshan Zheng, Guangyong Zhao, Linshu Jiang, Benhai Xiong

**Affiliations:** ^1^State Key Laboratory of Animal Nutrition, Institute of Animal Science, Chinese Academy of Agricultural Sciences, Beijing, China; ^2^State Key Laboratory of Animal Nutrition, College of Animal Science and Technology, China Agricultural University, Beijing, China; ^3^Beijing Dairy Cattle Center, Beijing Capital Agribusiness Group, Beijing, China; ^4^Beijing Key Laboratory for Dairy Cow Nutrition, Beijing University of Agriculture, Beijing, China

**Keywords:** non-forage fiber sources, methanogenesis, hydrogen metabolism, propionate production, archaeal and bacterial community

## Abstract

The rumen microbial complex adaptive mechanism invalidates various methane (CH_4_) mitigation strategies. Shifting the hydrogen flow toward alternative electron acceptors, such as propionate, was considered to be a meaningful mitigation strategy. A completely randomized design was applied in *in vitro* incubation to investigate the effects of replacing forage fiber with non-forage fiber sources (NFFS) in diets on methanogenesis, hydrogen metabolism, propionate production and the methanogenic and bacterial community. There are two treatments in the current study, CON (a basic total mixed ration) and TRT (a modified total mixed ration). The dietary treatments were achieved by partly replacing forage fiber with NFFS (wheat bran and soybean hull) to decrease forage neutral detergent fiber (fNDF) content from 24.0 to 15.8%, with the composition and inclusion rate of other dietary ingredients remaining the same in total mixed rations. The concentrations of CH_4_, hydrogen (H_2_) and volatile fatty acids were determined using a gas chromatograph. The archaeal and bacterial 16S rRNA genes were sequenced by Miseq high-throughput sequencing and used to reveal the relative abundance of methanogenic and bacterial communities. The results revealed that the concentration of propionate was significantly increased, while the concentration of acetate and the acetate to propionate ratio were not affected by treatments. Compared with CON, the production of H_2_ increased by 8.45% and the production of CH_4_ decreased by 14.06%. The relative abundance of *Methanomassiliicoccus* was significantly increased, but the relative abundance of *Methanobrevibacter* tended to decrease in TRT group. At the bacterial phylum level, the TRT group significantly decreased the relative abundance of *Firmicutes* and tended to increase the relative abundance of *Bacteroidetes*. The replacement of forage fiber with NFFS in diets can affect methanogenesis by shifting the hydrogen flow toward propionate, and part is directed to H_2_ *in vitro*. The shift was achieved by a substitution of *Firmicutes* by *Bacteroidetes*, another substitution of *Methanobrevibacter* by *Methanomassiliicoccus*. Theoretical predictions of displacements of H_2_ metabolism from methanogenesis to propionate production was supported by the dietary intervention *in vitro*.

## Introduction

Microorganisms in the rumen can ferment feeds rich in cellulose and can convert plant materials that people can’t utilize directly into meat and milk products. At the same time, the process of hydrolyzing complex compounds is accompanied by the formation of gasses, such as hydrogen (H_2_) and carbon dioxide (CO_2_). To keep the fermentation continuing, methanogenic archaea in the rumen produce methane (CH_4_) by using H_2_ and CO_2_ to scavenge H_2_ and keep the partial pressure of H_2_ low (Moss et al., 2000). Methanogenesis from ruminants can result in a significant loss of feed efficiency (2–12%), depending upon the types of diets (Johnson and Johnson, 1995). To mitigate the negative impact on climate change and to improve feed efficiency, various mitigation strategies have been conducted to reduce CH_4_ emissions from ruminants, including using essential oils (Patra and Yu, 2012), plant extracts (Wang et al., 2016), ionophores (Weimer et al., 2008), and vaccines (Williams et al., 2009). However, the rumen ecosystem is exceedingly complex and the ability of this system to efficiently convert complex carbohydrates to volatile fatty acids (VFA) is in part due to the effective disposal of H_2_ by reducing CO_2_ to produce CH_4_. Methanogenesis can be inhibited for short periods, but the ecology of the system is such that it frequently reverts back to the initial levels of methane production through all various adaptive mechanisms (McAllister and Newbold, 2008). On the other hand, the problem with chemical residues, toxicity, and high costs, have greatly limited these strategies utilization in animal production.

The type of feed offered to ruminants has a major effect on the profile of VFA and the level of methane production. Kessel and Russell (1996) reported that methane production was completely inhibited at a pH less than 6.0 when feeding a high-concentrate diet. However, when microorganisms in the rumen are exposed to large amounts of fermentable substrates for short periods, the rate of VFA production will exceed the VFA utilization, resulting in subacute ruminal acidosis or acute ruminal acidosis, which has negative impacts on animal health and performance (Nagaraja and Titgemeyer, 2007; Sato, 2015). Non-forage fiber sources (NFFS) from high-fiber byproducts usually have limited utility in non-ruminant diets, but ruminant nutritionists can use them to partially replace both forages and concentrates in lactation diets (Allen and Grant, 2000; Stock et al., 2000). Although they have different production responses (Huhtanen, 1993; Huhtanen et al., 1995; Alamouti et al., 2009), NFFS-based diets can maintain or improve the performance of dairy cattle under certain conditions (Pereira et al., 1999; Ertl et al., 2015). The strategy of primarily replacing some forage fiber with NFFS for higher-producing cows but only partially replacing some starch for lower-producing cows can optimize nutrient utilization and potentially control feed costs without compromising animal health or productivity (Bradford and Mullins, 2012). Moreover, Pereira and Armentano (2000) reported that low-forage, medium-neutral detergent fiber (NDF) (12.6% forage NDF, 27.5% total NDF) diets and low-forage, high-NDF diets (12.7% forage NDF, 35.7% total NDF) had a lower acetate to propionate ratio and a higher proportion of propionate than the traditional diet (20.1% forage NDF, 25.2% total NDF). The formation of acetate and butyrate results in production of H_2_ which can be used to generate CH_4_ by methanogenic archaea (Moss et al., 2000). Propionate is an end-product of rumen fermentation that is probably the principal alternative of the H^+^ sink after CH_4_, and the acetate to propionate ratio in the rumen has a relationship with methanogenesis (Lana et al., 1998; Russell, 1998). The balance between propionate formation and acetate and butyrate formation has a key role in determining H_2_ available in rumen for utilization by methanogenic archaea. Janssen (2010) reported that increases in propionate formation are strongly associated with decreases in CH_4_ production. In a meta-analysis, Ungerfeld (2015) found that inhibiting CH_4_ production in batch cultures resulted in redirection of metabolic hydrogen toward propionate and H_2_, but not butyrate. In addition, propionate is predominantly used as a glucose precursor in ruminants, and more propionate formation would likely result in a more efficient utilization of feed energy. Maximizing the flow of metabolic hydrogen in the rumen away from CH_4_ and toward VFA (mainly propionate) would increase the efficiency of ruminant production and decrease its environmental impact (Ungerfeld, 2015). Therefore, modifying the dietary formulation with NFFS may be an effective measure to shift the hydrogen flow toward alternative electron acceptors such as propionate.

The objective of this study was to build a model of metabolic hydrogen shifts *in vitro* to explore the effects of replacing forage fiber with NFFS in diets on methanogenesis, hydrogen metabolism, propionate production and the methanogenic and bacterial community by high-throughput sequencing.

## Materials and Methods

### Experimental Design and *in vitro* Incubation

A completely randomized design was applied in an *in vitro* incubation to investigate the effects of the replacement of forage fiber with NFFS in diets on rumen fermentation, methanogenesis and the methanogenic and bacterial community. There are two treatments in the current study: CON (a basic total mixed ration) and TRT (a modified total mixed ration). The dietary treatments were achieved by partly replacing forage fiber with NFFS (wheat bran and soybean hull) to decrease the content of forage neutral detergent fiber (fNDF) from 24.0 to 15.8%, with the composition and inclusion rate of other dietary ingredients remaining the same in total mixed rations (TMRs) (Table 1).

**Table 1:** Composition and nutrient levels of experiment substrates.

Item	Experiment Substrates	
	CON	TRT
**Ingredient, % of DM**		
Alfalfa hay	18.59	5.51
Corn silage	25.65	22.06
Steam-flaked corn	26.02	26.47
Soybean meal	7.43	7.35
Cottonseed meal	7.43	7.35
Beet pulp	5.58	5.51
DDGS	7.43	7.35
Wheat bran	–	8.27
Soybean hull	–	8.27
Premix^1^	1.86	1.84
**Chemical composition, % of DM**		
CP	16.4	16.4
RDP (% of CP)	56.3	56.7
NDF	34.2	34.5
f NDF	24.0	15.8
ADF	23.0	23.2
NFC^2^	39.3	35.6
Starch	24.9	24.8
NE_L_^3^, Mcal/kg	1.53	1.60

*^1^Premix composition per kilogram: 1,230 mg of Cu [minimum (min)], 4,950 mg of Zn (min), 1,760 mg of Mn (min), 50 mg of I (min), 61 mg of Se (min), 37 mg of Co (min), 504,800 IU of vitamin A (min), 88,800 IU of vitamin D_3_ (min), and 2,100 IU of vitamin E (min), 700 mg of vitamin B_3_ (min).*

*^2^Calculated as 100 − (% NDF + % CP + % ether extract + % ash).*

*^3^Estimated according to National Research Council [NRC] (2001)*.

Chinese Academy of Agricultural Sciences Animal Care and Use Committee (Beijing, China) approved the procedures used to collect the rumen fluid from donor animals. The ruminal fluid for the *in vitro* incubations was collected from three rumen-cannulated lactating Holstein cows 2 h after the morning feeding; the animals were fed a TMR twice daily. Ruminal fluid was brought to the laboratory within 30 min and was strained through layers of cheesecloth under continuous flushing with CO_2_. Equal volumes of rumen fluid collected from each of the cows were combined as the inoculum and diluted with buffer solution (1:2 v/v), which was prepared as described by (Menke, 1988), at 39°C with the continuous flow of CO_2_ for 3–5 s to remove headspace air. The inoculum-buffer mixture was dispensed into 120-ml serum bottles (75 mL/bottle) containing 500 mg of TMR substrates (CON or TRT). An extra three bottles without any substrate served as blanks. After they were sealed with butyl rubber plus crimped aluminum seals, all the serum bottles were connected with vacuumed air bags and incubated at 39°C for 48 h with horizontal shaking at 60 rpm. The *in vitro* incubation was repeated in three experimental batches and three fermentations per treatment were arranged in each batch.

### Sampling and Analyses of Biogas and VFAs

At the end of the 48 h of incubation, the total gas production of each air bag was measured using 100 mL calibrated glass syringes (Häberle Labortechnik, Lonsee-Ettlenschieß, Germany), and the inner wall was coated with Vaseline. The pH values of the *in vitro* incubations were measured using a portable pH meter (Seven Go^TM^ portable pH meter, Mettler Toledo, Switzerland). The whole biomass material in each bottle was individually filtered through pre-weighed nylon bags (8 cm × 12 cm, 42 μm). The filtered incubation fluid samples (2.5 ml) were individually collected into microcentrifuge tubes and preserved at −80°C for VFAs and microbial analysis. Afterwards, the nylon bags were washed gently with cold running water till the effluent ran clear and then they were dried at 55°C for 48 h till they achieved a constant weight for analysis of dry matter (DMD), neutral detergent fiber (NDFD) and acid detergent fiber (ADFD) disappearance. The contents of NDF and ADF were determined using the Ankom A200 fiber analyzer (Ankom Technology, Macedon, NY, United States) based on the procedures of Van Soest et al. (1991) using α-amylase and sodium sulfide for NDF.

The concentrations of methane and hydrogen in the gas samples were determined using a gas chromatograph (7890B, Agilent Technologies, United States) equipped with a thermal conductivity detector and a packed column (Porapak Q, Agilent Technologies, United States). The initial oven temperature was maintained at 30°C and held at this temperature for 1.5 min, and the reaction continued for 1.5 min. The detector temperature was 100°C, and the carrier gas was nitrogen. Gas production volume (total gas, CH_4_ and H_2_) was corrected for temperature (25°C) and pressure (101.325 kPa) conditions. The productions of methane and hydrogen were calculated from the methane and hydrogen concentration and total gas production. The VFA concentrations in the cultures were also analyzed by a gas chromatograph (7890B, Agilent Technologies, United States) fitted with a flame ionization detector and a capillary column (30 m × 0.250 mm × 0.25 μm; BD-FFAP, Agilent Technologies, United States). The oven initial temperature was maintained at 90°C, increased by 5°C/min to 120°C, holding at this temperature for 2 min, and the reaction continued for 8 min. Then, the temperature was increased by 20°C/min to 250°C and held at this temperature for 10 min and the reaction continued for 24.5 min. The injector temperature was 250°C, the detector temperature was 280°C, and the carrier gas was nitrogen.

### DNA Extraction

Microbial DNA was extracted using an extraction kit (E.Z.N.ATM Mag-Bind Soil DNA Kit, Omega, Norcross, Georgia, United States) in triplicate according to the manufacturer’s instructions and the resulting extracts were composited to average out the bias in sampling and extraction. The concentration and quality of the extracted DNA were assessed by 1% agarose gel electrophoresis and a Qubit 3.0 spectrometer (Invitrogen, United States), respectively.

### Microbial 16S rRNA Genes Amplification and Illumina Sequencing

The V4 region of the bacterial and archaeal 16S rRNA gene was amplified using the universal primers 515F (5′-GTGCCAGCMGCCGCGGTAA-3′) and 806R (5′- GGACTACHVGGGTWTCTAAT-3′) (Caporaso et al., 2011) with forward primers tagged with unique barcode sequences for each sample. While the abundance of the archaeal community was much lower in comparison to the bacterial community, the archaeal community was especially analyzed using nested PCR. In the nested PCR approach, the specific archaeal community was first amplified using the primers Arch340F (5′-CCCTAYGGGGYGCASCAG-3′) and Arch1000R (5′-GAGARGWRGTGCATGGCC-3′) as described by Gantner et al. (2011), and then the PCR product was used as a template in the second PCR using the primers Arch349F (5′-GYGCASCAGKCGMGAAW-3′) and Arch806R (50-GGACTACVSGGGTATCTAAT-3′) (Takai and Horikoshi, 2000). A 30 μL PCR reaction mixture contained 15 μL of 2 × Taq Master Mix, 1 μL of 10 μM Bar-PCR primer F, 1 μL of 10 μM Primer R, and 10–20 ng of genomic DNA template. PCR was performed using a T100^TM^ Thermal Cycler (BIO-RAD, United States) under the following conditions: 94°C for 3 min; followed by 5 cycles of 94°C for 30 s, 45°C for 20 s, and 65°C for 30 s; then 20 cycles of 94°C for 20 s, 55°C for 20 s, and 72°C for 30 s; and finished with a final extension at 72°C for 5 min. Illumina bridge-type compatible PCR primers were introduced for the next round (the second round for bacterial DNA and the third round for archaea DNA) of PCR. The next round of PCR was performed under the following conditions: 95°C for 3 min followed by 5 cycles of 94°C for 20 s, 55°C for 20 s, and 72°C for 30 s; and concluding with a final extension at 72°C for 5 min. DNA was amplified in triplicate for each sample, and then PCR amplicons were further purified with a DNA purification kit (Gel Purification kits, Sangon, Shanghai, China), and the concentrations were determined using spectrometry (Qubit 3.0, Invitrogen, United States). At last, the purified 16S rRNA gene amplicons were pooled and subjected to paired-end sequencing using the Illumina MiSeq platform at Shanghai Sangon Biotech Co., Ltd.

### Sequencing Data Processing and Analysis

Pairs of reads from the original DNA fragments were first merged using FLASH (Magoč and Salzberg, 2011), and then PRINSEQ was used for quality control of these merged reads (Schmieder and Edwards, 2011). Merged reads were assigned to each sample based on the unique barcode. Then, the barcode and primers were removed. PCR chimeras were filtered out using UCHIME (Edgar et al., 2011). After the above filtration, the average length of all the clean reads was 416 and 379 bp, and the average sequencing depth was ca. 68,381 and 61,343 clean reads for bacterial and archaeal community analysis, respectively. Operational taxonomic units (OTU) were clustered at 97% sequence identity using UPARSE (Edgar et al., 2011). The taxonomic classification of the sequences was carried out using the Ribosomal Database Project (RDP) Classifier at the bootstrap cutoff of 80% as suggested by the RDP. Simpson, Shannon, Chao1, Coverage and ACE indices were calculated for each sample. The weighted UniFrac distance was used for a principal coordinate analysis (PCoA) (Lozupone et al., 2007), and an analysis of similarity (ANOSIM) in QIIME with 999 permutations (R Core Team, 2013) was conducted to assess the significant differences between treatments. The relative abundance of bacteria was expressed as a percentage. The difference in relative abundance of bacteria and archaea was expressed as a percentage and extended error bar plot was performed to visualize the difference by bioinformatics software (STAMP). Welch’s two-sided test was used and Welch’s inverted was 0.95 (Parks et al., 2014).

### Statistical Analysis

Gas production volume (total gas, CH_4_ and H_2_), pH, VFAs, DMD, NDFD, ADFD, bacterial abundance, and diversity index were analyzed using PROC MIXED of SAS 9.4 (SAS Institute, Inc., Cary, NC, United States) as shown in the following model: *Y*_ijk_ = μ + T_i_ + B_j_ + TB_ij_ + e_ijk_, where *Y*_ijk_ is the dependent variable, μ is the overall mean, *T*_i_ is the effect of treatment (CON or TRT, considered fixed), *B*_j_ is the effect of batches (*j* = 1,2,3, considered fixed), TB_ij_ is the interaction between *T*_i_ and *B*_j_ (considered fixed effects), and *e*_ijk_ is the residual. Data on the bacterial abundance were transformed to log10 (n+1) to ensure normal distribution. Other data were checked for normal distribution and homogeneity by Shapiro–Wilk’s and Levene’s tests in SAS 9.4. Differences were declared significant at *P* < 0.05 and trends at 0.05 ≤*P* < 0.10.

### Nucleotide Sequence Accession Number

All the raw sequences were submitted to the NCBI Sequence Read Archive (SRA^1^), under accession number SRP134679.

## Results

### Biogas Production, Degradability, pH, and VFA

The DMD, NDFD, and ADFD increased significantly when the dietary forage fibers were partly replaced by NFFS. The concentration of propionate was significantly increased, while the concentration of the acetate and acetate to propionate ratio were not affected by the treatments. The production of total gas was not significantly affected in response to treatments, it just decreased by only 6.4%. Compared with CON, the production of H_2_ of TRT increased by 8.45% and was also not significantly affected. The production of CH_4_ tended to decrease by 14.06%, when the dietary forage fibers (mainly from alfalfa) were partly replaced by NFFS (wheat bran and soybean hull) (Table 2).

**Table 2:** Effects of the replacement of forage fiber with NFFS in diets on rumen fermentation and biogas production *in vitro* (*n* = 9).

Items	Treatment (f NDF/%)^1^		SEM^2^	*P*
	CON(24.0)	TRT(15.8)	
pH	6.73	6.72	0.006	0.3782
DMD^3^	0.8227	0.8450	0.00440	0.0007
NDFD^4^	0.7717	0.8296	0.00923	0.0007
ADFD^5^	0.7492	0.8169	0.01074	0.0005
TVFA/(mmol/L)^6^	77.14	77.56	0.792	0.6896
Acetate/(mmol/L)	47.72	47.91	0.563	0.7966
Propionate/(mmol/L)	17.90	18.30	0.140	0.0146
Isobutyrate/(mmol/L)	0.73	0.74	0.020	0.9023
Butyrate/(mmol/L)	8.57	8.43	0.119	0.5361
Isovalerate/(mmol/L)	1.32	1.28	0.038	0.4917
Valerate/(mmol/L)	0.90	0.90	0.021	0.8978
Acetate/Propinate	2.60	2.62	0.029	0.2347
Total gas production/(mL)	77.56	72.56	4.829	0.6258
H_2_/(μg)	11.37	12.42	0.481	0.2526
CH_4_/(mg)	6.26	5.38	0.232	0.0789

*^1^CON, control diet, a basic total mixed ration; TRT, treatment diet, a modified total mixed ration with replacement of alfalfa by wheat bran and soybean hull.*

*^2^SEM, standard error of the mean.*

*^3^DMD, dry matter disappearance.*

*^4^NDFD, neutral detergent fiber disappearance.*

*^5^ADFD, acid detergent fiber disappearance.*

*^6^TVFA, total valid fatty acid*.

### Diversity of Methanogenic and Bacterial Community

The structure of the archaeal and bacterial community was characterized by sequencing the V4 region of 16S rRNA gene with Illumina MiSeq.

#### Archaea

**FIGURE 1:**
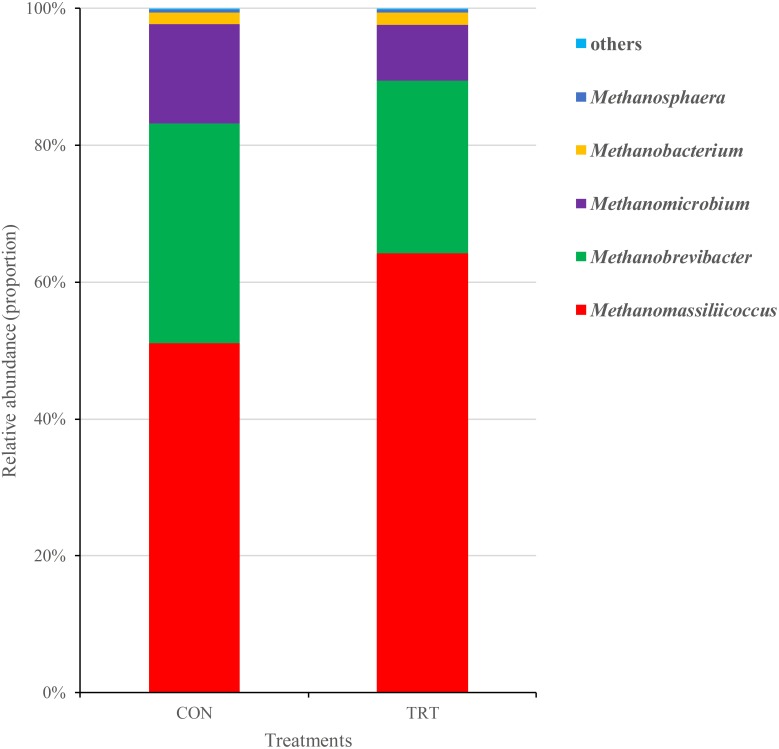
Composition of the predominant archaea genera among treatments *in vitro*. CON, control diet, a basic total mixed ration; TRT, treatment diet, a modified total mixed ration with the replacement of forage fiber by wheat bran and soybean hull. The top 5 abundant archaea genera are shown and the others are not shown (*n* = 9).

After merging and a quality check, a total of 1,182,452 merged sequences and 1,142,612 high-quality sequences were generated from 18 samples, with an average read length of 379 bases acquired. After filtering the chimeras, the remaining 1,104,189 sequences were used to generate OTUs with 97% sequence similarity across all the samples. The OTU table was filtered, leaving 5,163 OTUs for further analysis. The archaeal community was represented by two different phyla, *Euryarchaeota* and *Thaumarchaeota*, where *Euryarchaeota* represented on average 99.9%. Genera that were each represented by ≥0.1% of the total sequences were selected for further analysis. The 5 predominant genera were *Methanomassiliicoccus* (57.62%), *Methanobrevibacter* (28.67%), *Methanomicrobium* (11.36%), *Methanobacterium* (1.76%), and *Methanosphaera* (0.37%) (Figure 1).

**FIGURE 2:**
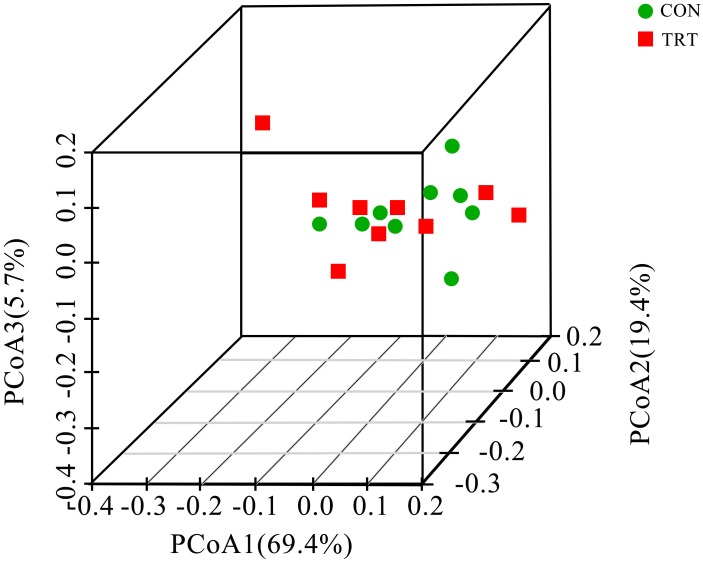
Principal coordinate analysis (PCoA) of archaeal community structures among treatments *in vitro*. PCoA plots were performed using the weighted UniFrac method. CON, control diet, a basic total mixed ration; TRT, treatment diet, a modified total mixed ration with the replacement of forage fiber by wheat bran and soybean hull (*n* = 9).

Alpha diversity indices of the archaeal community were presented in Table 3. No significant differences were observed among treatments based on the OTU numbers and coverage, indicating that the sequencing depth was desirable for the analysis. The alpha diversity indices of Chao1, ACE, Shannon and Simpson were not affected by treatments, showing that decreasing the content of fNDF from 24.0 to 15.8% didn’t change the archaeal community richness and diversity. PCoA analysis based on the weighted UniFrac metrics was performed to compare the two treatments (Figure 2). The ANOSIM analysis revealed no significant differences in the structure of the archaeal community between CON and TRT (*R* = 0.016, *P* = 0.304). Principal coordinate 1, 2, and 3 accounted for 69.4, 19.4, and 5.7% of the total variation, respectively.

**Table 3:** Alpha diversity indices of archaea and bacteria among treatments *in vitro* (*n* = 9).

Items	Treatment (f NDF/%)^1^		SEM^2^	*P*
	CON(24.0)	TRT(15.8)	
**Archaea**			
OTU^3^	1127	1093	25.0	0.5057
Coverage	0.99	0.99	0.001	0.3506
Chao1	2042	1888	46.7	0.0946
ACE^4^	2631	2625	171.6	0.9890
Shannon	2.30	2.25	0.030	0.3591
Simpson	0.25	0.27	0.009	0.0951
**Bacteria**				
OTU	4062	4131	54.3	0.5868
Coverage	0.98	0.98	0.001	0.7395
Chao1	5978	5983	79.1	0.9802
ACE	6926	6787	134.2	0.6396
Shannon	6.46	6.51	0.019	0.0954
Simpson	0.005	0.005	0.0001	0.5094

*^1^CON, control diet, a basic total mixed ration; TRT, treatment diet, a modified total mixed ration with replacement of forage fiber by wheat bran and soybean hull.*

*^2^SEM, standard error of the mean.*

*^3^OTU, operational taxonomic units.*

*^4^ACE, abundance-based coverage estimator*.

#### Bacteria

**FIGURE 3:**
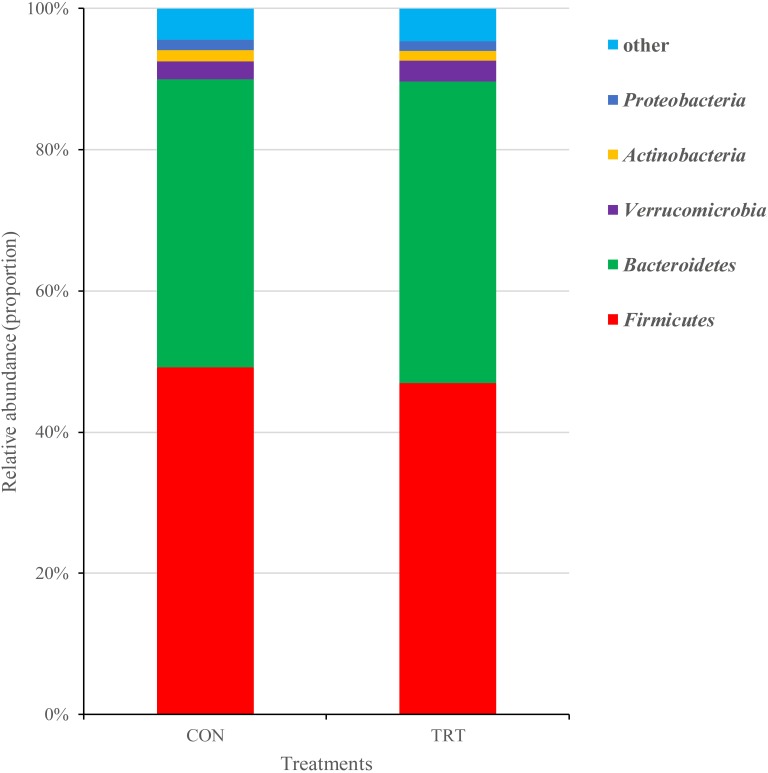
Composition of the predominant bacterial phyla among treatments *in vitro*. CON, control diet, a basic total mixed ration; TRT, treatment diet, a modified total mixed ration with the replacement of forage fiber by wheat bran and soybean hull. The top 5 abundant bacteria phyla are shown and the others are not shown (*n* = 9).

A total of 1,414,889 merged sequences and 1,367,883 high-quality sequences were generated from 18 samples, with an average read length of 416 bases acquired. After removing chimeric sequences, the remaining 1,230,862 sequences were used to generate OTUs with 97% sequence similarity across all the samples. After filtering the OTU table, 15,542 OTUs were left for further analysis. Across all samples, 19 bacterial phyla were identified. *Firmicutes* and *Bacteroidetes* were the dominant phyla, representing 48.03 and 41.79% of the total sequences, respectively. *Verrucomicrobia*, *Actinobacteria*, and *Proteobacteria* represented average percentages of 2.74, 1.47 and 1.44%, respectively (Figure 3). The remaining phyla represented less than 1% of all the sequences. Genera that were each represented by ≥0.1% of the total sequences were selected for further analysis. The nine predominant genera were *Prevotella* (6.59%), *Acetobacteroides* (3.87%), *Falsiporphyromonas* (3.60%), *Succiniclasticum* (3.20%), *Sporobacter* (2.68%), *Subdivision5_genera_incertae_sedis* (2.31%), *Ruminococcus* (2.21%), *Lachnospiracea_incertae_sedis* (2.12%) and *Lachnobacterium* (2.08%).

**FIGURE 4:**
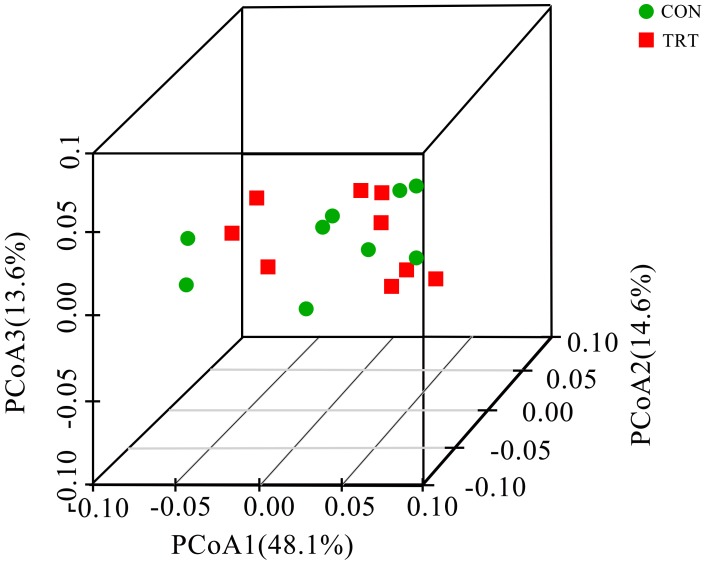
Principal coordinate analysis (PCoA) of bacterial community structures among treatments *in vitro*. PCoA plots were performed using the weighted UniFrac method. CON, control diet, a basic total mixed ration; TRT, treatment diet, a modified total mixed ration with the replacement of forage fiber by wheat bran and soybean hull (*n* = 9).

Alpha diversity indices of the bacterial community were also presented in Table 3. Similar to the archaeal community, no significant differences were observed about any alpha diversity indices. Therefore, the replacement of forage fiber with NFFS in diets didn’t change the bacterial community richness and diversity. PCoA analysis based on the weighted UniFrac metrics was performed to compare the two treatments (Figure 4). The ANOSIM analysis revealed no significant differences on the structure of the bacterial community between treatments (*R* = −0.100, *P* = 0.953). Principal coordinate 1, 2, and 3 accounted for 48.1, 14.6, and 13.6% of the total variation, respectively.

### Relative Abundance of Methanogenic and Bacterial Communities

#### Archaea

**FIGURE 5:**
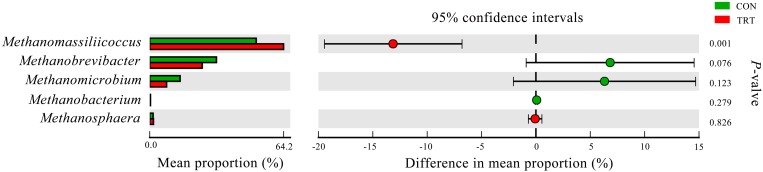
Difference in the relative abundance of 5 predominant archaeal genera (abundance of the genera was expressed as %). Extended error bar plot was performed by bioinformatics software (STAMP). Welch’s two-sided test was used and Welch’s inverted was 0.95. CON, control diet, a basic total mixed ration; TRT, treatment diet, a modified total mixed ration with the replacement of forage fiber by wheat bran and soybean hull (*n* = 9).

At the phylum level, *Euryarchaeota*, as the most predominant archaeal phylum, represented on average 99.9% of the all sequences. *Methanomassiliicoccus*, *Methanobrevibacter*, *Methanomicrobium*, *Methanobacterium*, and *Methanosphaera* from *Euryarchaeota* were the five predominant genera in the present study. The relative abundance of *Methanomassiliicoccus* was significantly increased in TRT compared with the CON group (*P* = 0.001). However, the relative abundance of *Methanobrevibacter* tended to decrease in the TRT group (*P* = 0.076). Similarly, the relative abundance of *Methanomicrobium* was lower in TRT group, but not significantly decreased (*P* = 0.123) (Figure 5).

#### Bacteria

**FIGURE 6:**
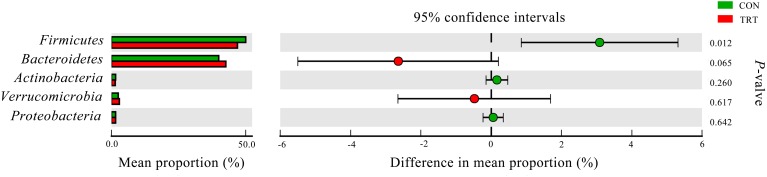
Difference in the relative abundance of 5 predominant bacteria phyla (abundance of the phylum was expressed as %). Extended error bar plot was performed by bioinformatics software (STAMP). Welch’s- two-sided test with two-side was used and Welch’s inverted is was 0.95. CON, control diet, a basic total mixed ration; TRT, treatment diet, a modified total mixed ration with the replacement of forage fiber by wheat bran and soybean hull (*n* = 9).

At the phylum level, replacement of the forage fiber with NFFS in diets significantly decreased the relative abundance of *Firmicutes* (*P* = 0.012) and tended to increase the relative abundance of *Bacteroidetes* (*P* = 0.065). The other phylum that accounted for ≥1% of the total sequences in at least one of the samples were not significantly affected by treatments (Figure 6).

**FIGURE 7:**
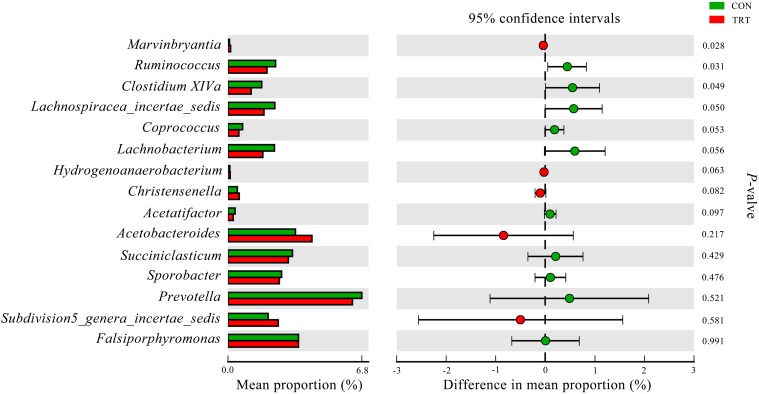
Difference in the relative abundance of bacterial genera that accounted for ≥0.1% of the total sequences in at least one of the samples and other predominant genera (abundance of the genera was expressed as %). Extended error bar plot was performed by bioinformatics software (STAMP). Welch’s two-sided test was used and Welch’s inverted was 0.95. CON, control diet, a basic total mixed ration; TRT, treatment diet, a modified total mixed ration with the replacement of forage fiber by wheat bran and soybean hull (*n* = 9).

At the genus level, the relative abundance that was represented by ≥0.1% of the total sequences in at least one of the samples were further analyzed. The influenced genera by treatments and the other six predominant genera were listed in Figure 7. All the influenced genera belonged to *Firmicutes*. Compared with the CON group, the relative abundance of most influenced genera was decreased. Specifically, replacement of forage fiber with NFFS in diets significantly decreased the relative abundance of *Ruminococcus* (*P* = 0.031) and *Clostridium XIVa* (*P* = 0.049) and tended to decrease the relative abundance of *Lachnospiracea_incertae_sedis* (*P* = 0.050), *Coprococcus* (*P* = 0.053), *Lachnobacterium* (*P* = 0.056) and *Acetatifactor* (*P* = 0.097). In contrast, the relative abundance of *Marvinbryantia* (*P* = 0.08) was significantly increased in the TRT group. The relative abundance of *Hydrogenoanaerobacterium* (*P* = 0.063) and *Christensenella* (*P* = 0.063) were tended to increase in TRT group. In addition, the relative abundance of *Succiniclasticum*, *Sporobacter* and *Prevotella* was higher, but the relative abundance of *Acetobacteroides* and *Subdivision5_genera_incertae_sedis* was lower in the TRT group. They were not significantly affected by treatments.

## Discussion

Non-forage fiber sources are generated by several industries, which are high in fiber (like forages) but are rapidly passed from the rumen (like concentrates). The judicious use of NFFS can improve the productivity and health of cattle in all stages of lactation while potentially controlling feed costs (Bradford and Mullins, 2012). Most non-forage fiber is relatively digestible compared with forge fiber, resulting in higher digestibility (Bhatti and Firkins, 1995; Dann et al., 2007). In our study, the replacement of forage fiber with NFSS significantly increased DMD, NDFD, and ADFD, but numerically increased the concentration of TVFA. On the contrary, total gas production was numerically decreased, unlike the concentration of TVFA. This may be explained by the fact that non-forage fiber is more digestible, but not better in fermentation than alfalfa fiber, which usually was considered to be the best forage fiber. The production of CH_4_ decreased by 14.06%, and H_2_ production was numerically increased by 8.45%, followed by significant increases in the concentration of propionate. The formation of acetate and butyrate results in production of H_2_ which can be used to generate CH_4_ by methanogenic archaea (Moss et al., 2000). In propionate formation, pyruvate is reduced to propionate, while in H_2_ formation, protons (H^+^) are reduced to H_2_. The two pathways are both electron accepting, so Janssen (2010) thought that propionate formation was an alternative pathway to H_2_ formation and was accompanied by decreasing in CH_4_ production. The balance between propionate formation and acetate and butyrate formation has a key role in determining H_2_ available in rumen for utilization by methanogenic archaea. These results indicated that the replacement of forage fiber with NFFS may reduce the utilization of hydrogen in methanogenesis and shift hydrogen flow from CH_4_ to propionate and H_2_ by changing the relative abundance of certain archaea or bacteria. Similar to previous studies, inhibition of CH_4_ production in batch cultures resulted in the redirection of metabolic hydrogen toward propionate and H_2_ (Ungerfeld, 2015). Mitsumori et al. (2012) found that some [H] spared from CH_4_ production is redirected to propionate, and part is directed to atypical [H] sinks like H_2_.

As the sole producers of methane in the rumen, a correlation between the number of methanogens and methanogenesis might be expected. Wallace et al. (2014) and Veneman et al. (2015) suggested that the number of archaea, rather than the population structure, might be the major driver of methane production in the rumen. Some strategies, including supplementation of essential oils (Duarte et al., 2017b) or ionophores (Shen et al., 2017), reduce enteric methane emissions by inhibiting the activity or richness of the microbiota. Inhibition of the microbiota generally results in decreasing feed digestion (Ungerfeld, 2015). However, Morgavi et al. (2010) reported that decreasing methane production did not affected fiber digestibility in several *in vitro* experiments. Shen et al. (2017) found that supplementation of nisin in diets decreased methane production, while feed digestion was unaffected. This may be explained by shifts in the microbial population. Various methanogen groups have different methanogenic potential (Leahy et al., 2013) and a shift in the methanogen community toward less effective methanogenesis might also explain the differences in methane production. We observed decreased methane production and increased propionate concentration, while dry matter disappearance was increased in the present study. It is possible that changes in the population structure of the microbiota can also affect the methane production, like the inhibition of microbial richness or activity. Belanche et al. (2016) found that ivy fruit saponins reduced the methane production by modifying the structure of the methanogen community and decreasing its diversity. In contrast, chitosan promoted a shift in the fermentation pattern toward propionate production to reduce the methane production, which is achieved by a simplification of the structure in the bacterial community. Therefore, both the richness of the methanogen and the population structure of the microbial community plays an important role in methanogenesis. On the other hand, the richness and diversity of the archaeal and bacterial community were not affected by treatments, together with a similar pH value and TVFA production, also suggesting that the replacement of forage fiber with NFFS in the diets didn’t have significant detrimental effects on the overall rumen fermentation *in vitro*.

### Archaea

Methane is produced in the rumen as a product of normal fermentation of the feedstuffs, and methanogens, which belong to the domain *Archaea* and the phylum *Euryarchaeota* are the only known microorganisms capable of methane production (Hook et al., 2010). However, compared to the number of methanogens, the efficiency of different methanogens is considered to be more important in methanogenesis (Shi et al., 2014). Given that the positive relationship of increased methane production and increased transcripts of *mcrA* gene, methanogens that can express more *mcrA* genes are believed to contribute more to methane production (Freitag and Prosser, 2009). In our study, differences between treatments were observed at the genus level, where the relative abundance of *Methanomassiliicoccus* (belongs to *Methanomassiliicoccaceae* family) was significantly increased, but the relative abundance of *Methanobrevibacter* tended to decrease in the TRT group. Danielsson et al. (2017) reported that unclassified *Methanomassiliicoccaceae* was 1.5-fold more abundant in low CH_4_ emitters than that in high CH_4_ emitters. Luo et al. (2017) reported that dietary pea fiber increased the diversity of the colonic methanogen community structure of pigs with a shift from *Methanobrevibacter* to *Methanomassiliicoccus* and *Methanomassiliicoccus*-like genus. Hydrogenotrophic pathway, methylotrophic pathway and acetoclastic pathway are the three major pathways of methanogenesis. *Methanobrevibacter* is one kind of hydrogenotrophic methanogen, that converts H_2_ and/or formate to CH_4_ (Leahy et al., 2013), while *Methanomassiliicoccus*, which belong to the novel order *Methanomassiliicoccales*, has capacity to use methylamine substrates for methanogenesis by H_2_-dependent methylotrophic pathway (Lang et al., 2015; Moissl-Eichinger et al., 2018). Although methanogenic archaea can acquire substrates form environment, some species would increase efficiency by forming contacts with protozoa, which produce large quantities of H_2_ by their hydrogenosomes (Embley et al., 2003). *Methanobrevibacter* is considered to be the predominant protozoa-associated methanogens (Belanche et al., 2014), while the relation between *Methanomassiliicoccus* and protozoa has not been reported. Compared with *Methanobrevibacter*, *Methanomassiliicoccus* may be less effective in methane production. Different sources of fiber may stimulate the growth of different microbiota and change the relative abundance of the microbial community. Replacement of forage fiber with NFFS in diets would mitigate CH_4_ emission from ruminants by changing the relative abundance of *Methanobrevibacter* and *Methanomassiliicoccus* at the archaea level.

*Methanomassiliicoccus* is more abundant in our study than *Methanobrevibacter*, which was believed to be the most predominant genus in a previous study (Janssen and Kirs, 2008). In another recent study (Danielsson et al., 2017), *Methanobrevibacter* was also observed as the most predominant genus. Danielsson et al. (2017) used the same primers of both bacteria and archaea to construct 16S rRNA amplicon libraries, resulting in an average of 505 archaeal sequences per sample (61434 archaeal sequences per sample in our study). Different sequencing depths may be responsible for the difference between our study and others. Similar to our study, Paul et al. (2015) also reported a high abundance of *Methanomassiliicoccaceae* present in the rumen of Nili-Ravi buffalo by 16S rDNA analysis of archaea.

### Bacteria

In the present study, the rumen microbiota was dominated by *Bacteroidetes* and *Firmicutes*, which are considered to be the predominant phyla in most studies (Kim et al., 2010; de Menezes et al., 2011; Martinez-Fernandez et al., 2016; Duarte et al., 2017a). The *Bacteroidetes* are considered net H_2_ utilizers whereas the *Firmicutes* phylum includes H_2_ producers (Stewart et al., 1997). Belanche et al. (2016) reported that supplementing chitosan in the control diet promoted a shift in the fermentation pattern toward propionate production, which explained about a third of the decrease in methanogenesis, which was achieved by the substitution of fibrolytic (*Firmicutes* and *Fibrobacteres*) by amylolytic bacteria (*Bacteroidetes* and *Proteobacteria*). In another study, a shift in the microbiota with an increase in the *Bacteroidetes* to *Firmicutes* ratio was accompanied by a 30% decrease in methanogenesis and increase in propionate production, using chloroform as a methane inhibitor (Martinez-Fernandez et al., 2016). Kittelmann et al. (2013) observed a positive correlation between the occurrence of methanogens and fibrolytic bacteria. In the present study, the TRT group had a higher abundance of *Bacteroidetes* (mainly amylolytic bacteria) and a lower abundance of *Firmicutes* (mainly fibrolytic bacteria) compared with the CON group. This substitution of fibrolytic by amylolytic bacteria may result in the higher concentration of propionate as fermentation products in the TRT group.

At the genus levels, all bacterial genera whose relative abundance changed (*P* < 0.1) belonged to the *Firmicutes* phylum. Most of these genera have higher relative abundance in the CON group, except *Marvinbryantia*, *Hydrogenoanaerobacterium*, and *Christensenella*. *Marvinbryantia* belongs to the *Lachnospiraceae* family, which degrades complex polysaccharides to short chain fatty acids, including acetate, butyrate, and propionate (Biddle et al., 2013). *Hydrogenoanaerobacterium* belong to the *Ruminococcaceae* family, which contains a large number of healthy gut-associated butyrate-producing bacteria (Morissette et al., 2017). A recent study suggests that the presence of *Christensenella* in the gut, a low abundant (less than 0.001%) and highly heritable (transmissible from parent to offspring) bacterial genus, decreases body weight gain in obese mice (Goodrich et al., 2014). There are few references about these genera and whether they participate in the metabolism of propionate is unknown. However, as described above, these genera are all involved in energy metabolism and maybe have certain relationships with shifting hydrogen flow toward propionate.

## Conclusion

Theoretical predictions of displacements of H_2_ metabolism from methanogenesis to propionate production was supported by the dietary intervention *in vitro*. A modified dietary formulation strategy can affect methanogenesis by shifting the hydrogen flow toward propionate and partially toward to H_2_. The shift was achieved by a substitution of *Firmicutes* by *Bacteroidetes* and another substitution of *Methanobrevibacter* by *Methanomassiliicoccus*. In conclusion, the replacement of forage fiber with NFFS in diets may be a meaningful strategy to shift the hydrogen flow toward propionate and further studies need to be conducted to explore if the same microbiota modulation would be observed *in vivo*.

## Author Contributions

KW, BX, and LJ designed the study. KW, XN, SZ, and KC conducted the experiments. KW, XN, and LY analyzed the data. KW wrote the manuscript. JT, XN, GZ, LJ, and BX revised the paper. All authors carefully read and agreed to be accountable for all aspects of the work.

## Conflict of Interest Statement

The authors declare that the research was conducted in the absence of any commercial or financial relationships that could be construed as a potential conflict of interest.

## Footnotes

1 http://www.ncbi.nlm.nih.gov/Traces/sra/
